# Presence of Paradoxical Mid-Systolic Longitudinal Strain (Stretching) with Speckle Tracking in the Basal Inferior-to-Lateral Segments in Mitral Valve Prolapse †

**DOI:** 10.3390/jcdd13100518

**Published:** 2026-10-14

**Authors:** Antonio Auriti, Lucio Monaco, Elisa Rauseo, Lanfranco Antonini, Furio Colivicchi

**Affiliations:** 1 Lab of Clinical Advanced Echocardiography, Division of Clinic and Rehabilitative Cardiology, San Filippo Neri Hospital, 00135 Rome, Italy; 2 Division of Clinic and Rehabilitative Cardiology, San Filippo Neri Hospital, 00135 Rome, Italy; 3 Emergency Medicine, Azienda Ospedaliera San Giovanni Addolarata, 00184 Rome, Italy

**Keywords:** mitral valve prolapse, strain, speckle tracking, ventricular arrhythmias in mitral valve prolapse

## Abstract

Background and Purpose: Abnormality in the motion of the inferior papillary muscle was found in an early study of mitral valve prolapse (MVP). We visually observed an abnormal movement not only in the inferior wall but also in the anterolateral and inferolateral basal walls in several patients with MVP. Therefore, the aim of this work was to analyze these findings using strain (Speckle Tracking—STE). Methods: A total of 60 MVP patients having a superior systolic displacement of the posterior leaflet, in whole or in part of the scallops, of at least 2 mm, and 20 normal subjects as controls were included. Longitudinal strain was calculated in the three apical views (Philips iE 33 Matrix—implemented strain software). The strain in the three abovementioned basal segments was measured at 160 ms from the beginning of systole (mid-systole), and then we averaged their values to form a “triad” of averaged values that could be subsequently compared among the groups for statistical calculations. The Global Longitudinal Strain (GLS) was also calculated for all subjects. The Mann–Whitney U-test was used for comparisons. A “*p*” value ≤ 0.05 was considered significant. Results: According to the strain patterns, we were able to identify two distinct groups in MVP patients: Group 1 had a “paradoxical” strain at 160 ms (=positive, that means “mechanical stretching”) in at least a single segment of the aforementioned “triad” of basal segments (*n* = 35), and Group 2 had not a “paradoxical” strain (*n* = 25) in any of the aforementioned “triad” segments at the same mid-systolic point. Statistical significance was found between Group 1 and Group 2 at 160 ms and at the peak (*p* < 0.0001 for both) and the same was found for GLS (*p* < 0.01). Group 1 vs. controls, as expected, displayed a significant difference in the strain at 160 ms (*p* < 0.0001) while Group 2 vs. controls did not differ significantly, even at the peak. Conclusions: According to our results, we found in our MVP population (more than half of the patients) that some MVP patients have a “paradoxical” strain (=mechanical stretching) in at least a single segment of the basal inferior-to-lateral segments (that we have named the “triad”) in mid-systole, whereas others do not. The passive continuous stretching of myocardial cells is known to lead, with time, to the replacement of cells by fibrosis (and fibrosis is a condition that is well known to be a source of arrhythmias, and even malignant). Previous Cardiac Magnetic Resonance (CMR) studies already reported the presence of fibrosis in the same basal wall segments in some MVP patients. Thus, considering our strain results, in MVP patients, but only in those with ventricular arrhythmias, it is indicated, in our opinion the strain should be analyzed, as it is an inexpensive tool. If the strain is found to be “paradoxical” even in a single segment of what we have named the “triad”, a further evaluation with CMR (even if expensive) is recommended to look for the presence of fibrosis. Strain analysis is indicated because these findings may have implications for prognosis, further evaluation of arrhythmias and the selection of appropriate therapy.

## 1. Introduction

Mitral valve prolapse (MVP) is a relatively frequent finding with a prevalence in the general population ranging from 0.6% to 2.4%, and up to 5–10% according to many investigations. Patients with MVP show a very heterogeneous clinical spectrum [1], varying from asymptomatic status to many cardiac complications: several degrees of mitral regurgitation (MR), cardiac rhythm disturbances ranging from simple premature supraventricular beats or atrial fibrillation or premature ventricular beats or ventricular tachycardia and, rarely, even sudden cardiac death (SCD) [2,3].

MVP can present itself with a wide spectrum of valve changes: from an insignificant systolic superior displacement of one or more scallops or a part of them to more significant modifications, like leaflet thickening, elongated cords, and severe thickening and huge superior displacement of the leaflets due to myxomatous degeneration, along with several degrees of mitral regurgitation.

The mitral valve is a complex anatomical structure that includes not only the valve itself, but also the annulus, the papillary muscles, and the myocardium beneath the annulus: all the above structures contribute to the normal function of the mitral apparatus. Although the leaflets are the most commonly involved structures, MVP may involve any of the aforementioned components of the mitral apparatus and particularly, as we investigated, the ventricular wall beneath the valve. A very interesting former study described an abnormal traction of the inferior papillary muscle in MVP, postulating that such a finding could be related to the “mechanical and electrophysiological changes” in the left ventricle (LV) [4]. Indeed, in our daily clinical practice, we visually observed, in several patients, an odd movement of the basal segments, not only in the inferior but also in the anterolateral and inferolateral segments, all located beneath the posterior part of the mitral annulus.

Therefore, the aim of our study was to investigate this abnormal wall motion in greater depth by using 2D strain, specifically Speckle Tracking (STE), and the possible implications, as a simple hypothesis, of the relationship between STE and arrhythmias, as previously suggested.

## 2. Materials and Methods

The study was conducted in accordance with the Helsinki Declaration, and verbal informed consent to participate was obtained from all of the patients.

Echocardiography for strain analysis in patients and controls was recorded in all the 3 apical views, provided that the images were of good quality. Thus, we consecutively enrolled 60 MVP subjects (defined as a superior displacement of at least 2 mm in the inferior leaflet in one or more scallops—a few patients had both anterior and inferior displacement). Then, 20 normal subjects formed the control group. We excluded patients with the following: (1) moderate or severe MR, (2) global wall motion abnormalities, (3) known or suspected coronary artery disease, (4) patients with more than mild abnormalities of the other valves that could affect the left ventricle (LV) strain, (5) patients with more than mild diastolic dysfunction, (6) patients having pericardial disease, (7) subjects using cardio-toxic drugs, (8) presence of systemic disorders which might result in direct cardiac complications (such as diabetes, cancer under therapy, kidney failure, clinical obstructive/restrictive respiratory diseases, thyroid disease, autoimmune diseases), (9) patients with mitral-annular disjunction (MAD) which could interfere with the dynamics of the basal wall.

The control group was composed of subjects without MVP who met the same exclusion criteria, mostly coming from the blood donation clinic (Table 1).

All patients underwent 2D standard echocardiography with a Philips iE33 Matrix machine (Eindhoven, The Netherlands). STE analysis was performed by using the implemented software in the machine with the standard settings (version 9.0). Longitudinal STE was calculated in the 3 apical views: 2-chamber (2C), 3-chamber (3C), and 4-chamber (4C). After the initial semiautomatic processing, we manually carefully adjusted the region of interest (ROI) to encompass the entire thickness of the myocardium throughout the whole cardiac cycle. As we observed that one or more of the basal inferior (BI), basal anterolateral (BAL) and basal inferolateral (BIL) walls were involved in this altered wall movement, we averaged their values for statistical analysis and called these 3 averaged segments a “triad”. So, to test our hypothesis, we registered the longitudinal strain (LS) in each basal segment of the “triad”, in each patient, at 160 ms from the beginning of systole (mid-systole), and then we averaged the values to form the groups. Subsequently, we compared the averaged strain value at 160 ms and also the Global Longitudinal Strain (GLS) among the groups.

We chose the cut-off of 160 ms because it was the best point of the mid-systole in our population according to the mean heart rate and STE software in the machine (version 9.0).

### Statistics

Normally distributed variables are presented as mean ± SD. Statistical analysis was performed using the SPSS-22 software. Differences among groups were assessed using the Mann–Whitney U-test. A *p* value ≤ 0.05 was considered statistically significant.

## 3. Results

**Figure 1:**
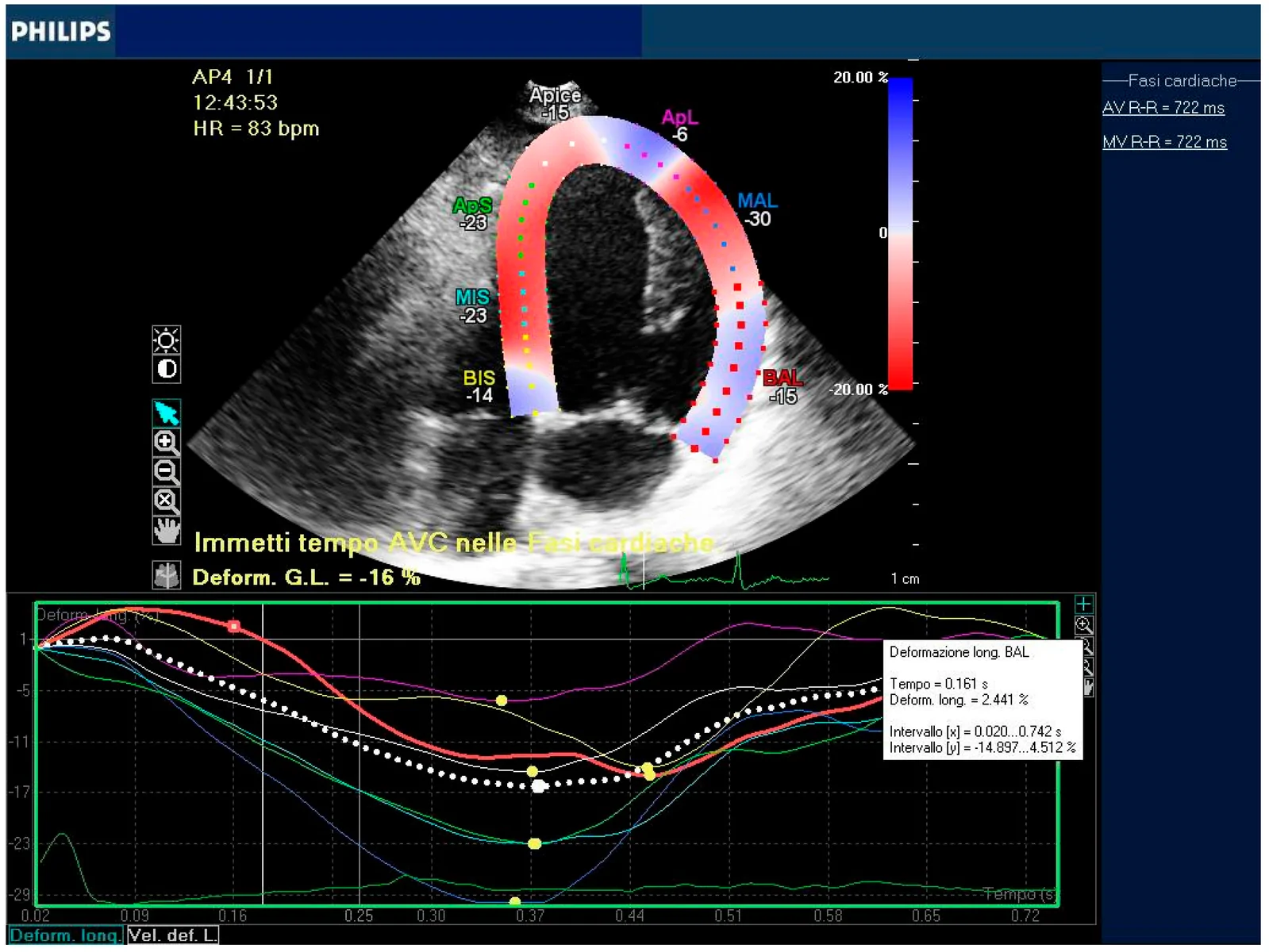
Example of a paradoxical strain in MVP (Group 1). 4C. At 160 ms the red thick line of BAL shows a positive value (2.44 = stretching).

**Figure 2:**
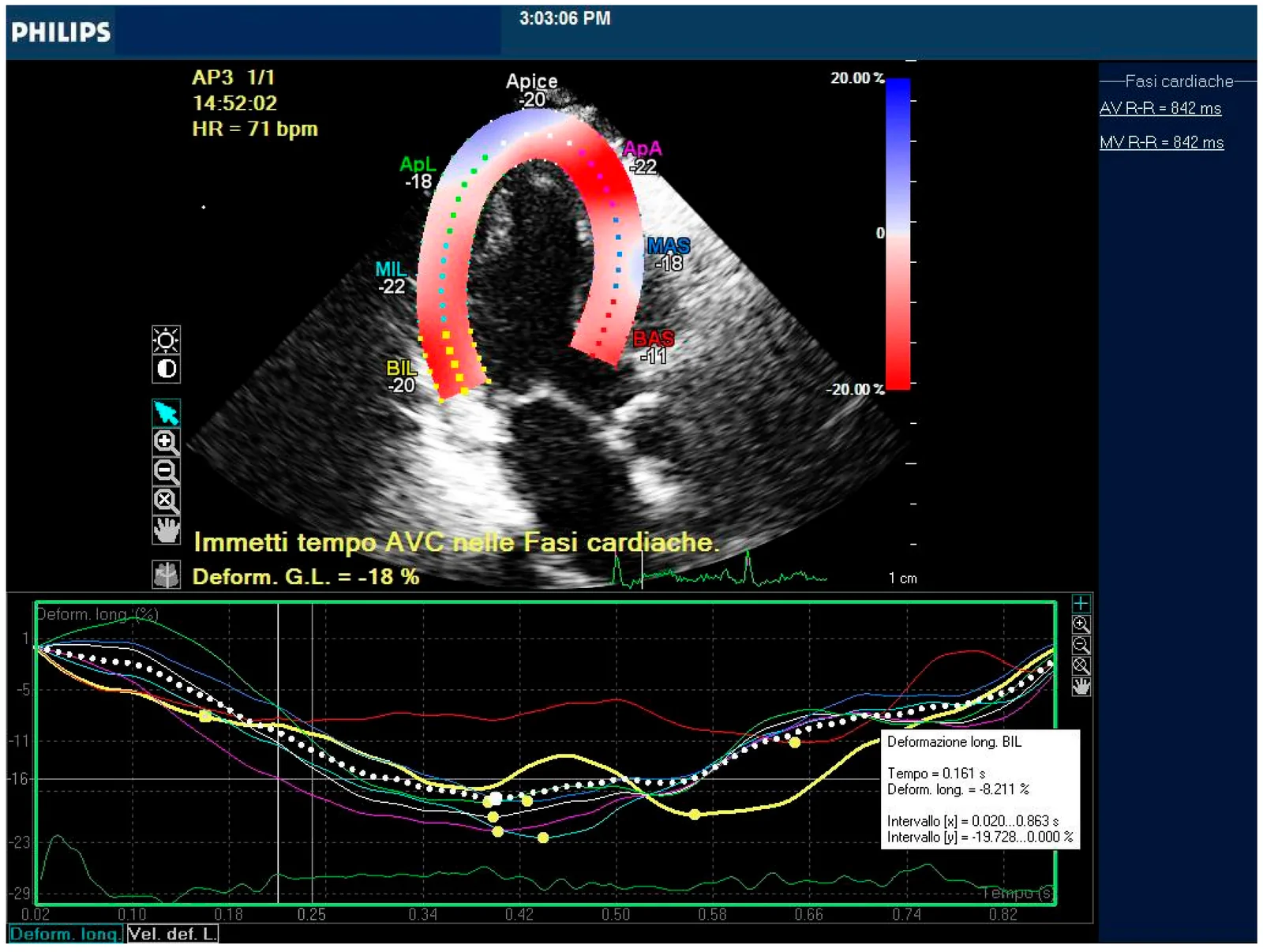
Example of a non-paradoxical strain in MVP (Group 2). 3C. At 160 ms the yellow thick line of BIL shows a normal negative value −8.21 = no stretching.

**Figure 3:**
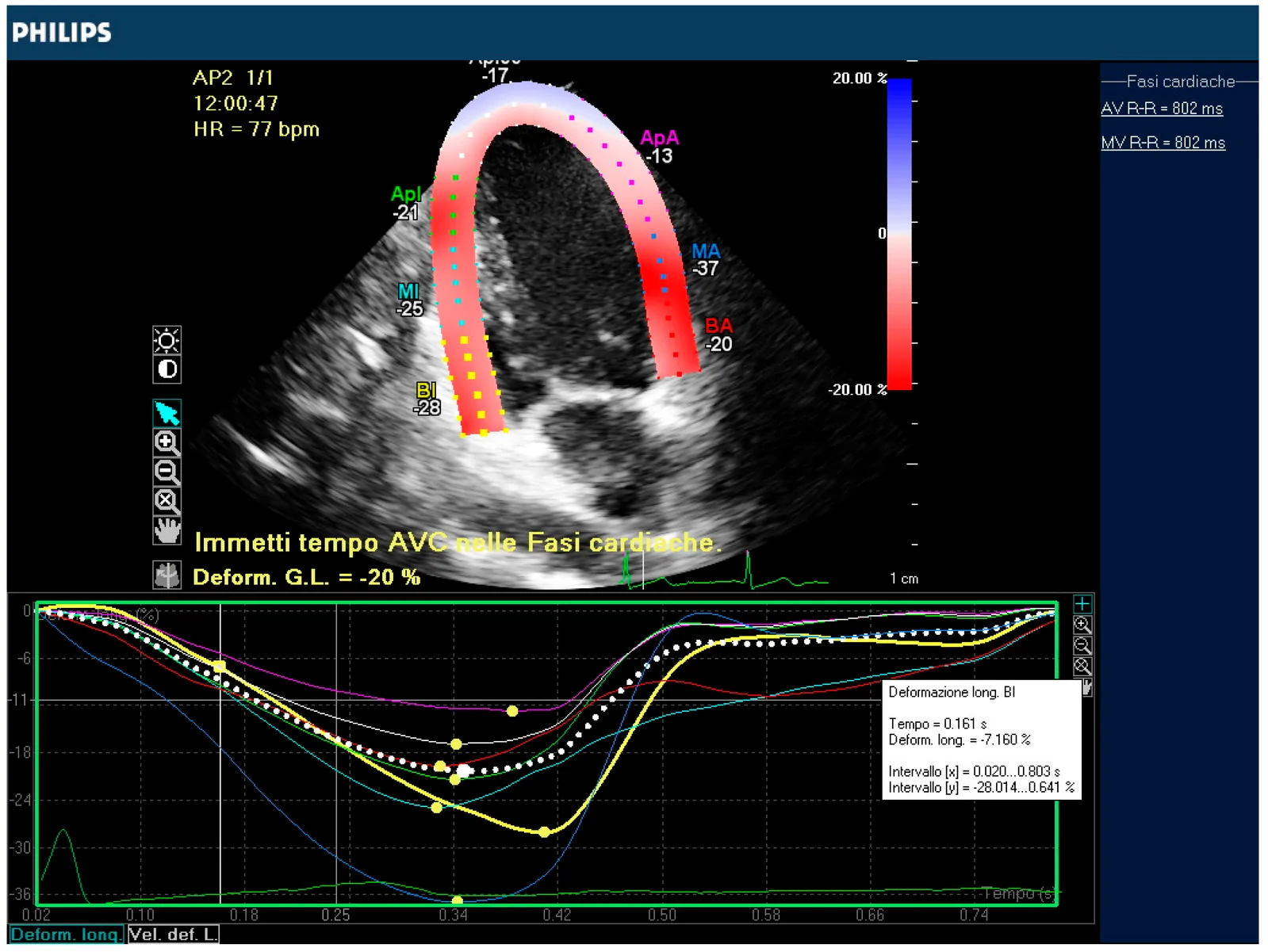
Example of a normal strain in one of the control subjects in 2C. At 160 ms the thick yellow line of BI shows a normal negative value (−7.16), and the same negative STE values were found in all the segments of the “triad”.

**Figure 4:**
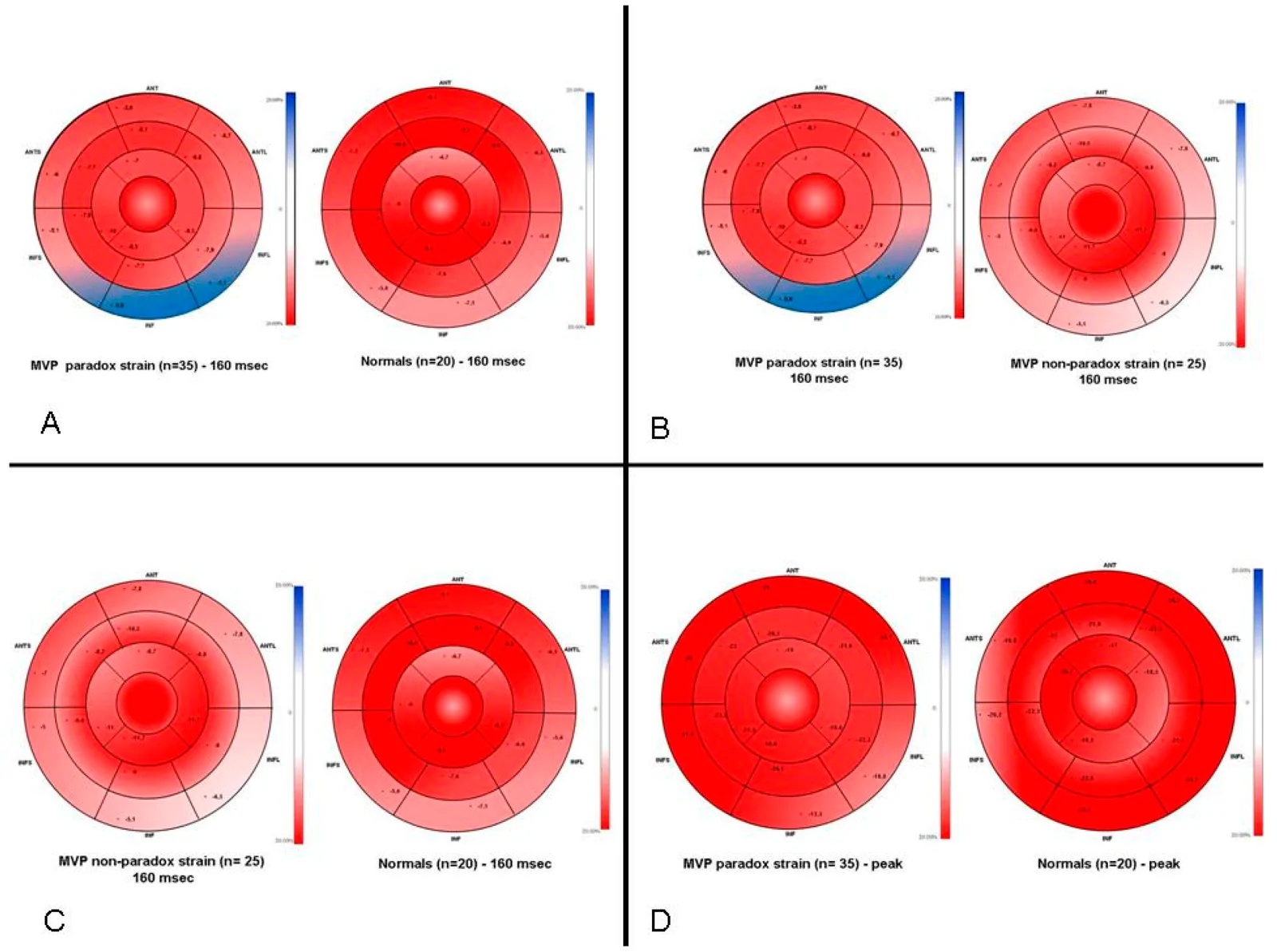
Bullseye view of strain in the different groups: panel: panel (**A**): MVP paradoxical strain vs. normals at 160 ms; panel (**B**): MVP paradoxical strain vs. non-paradoxical strain at 160 ms; panel (**C**): MVP non-paradoxical strain vs. normals at 160 ms; panel (**D**): MVP paradoxical strain vs. normals at peak; (blue paradoxical strain).

According to our results in STE, we were able to recognize, in MVP patients of our population, two groups having different strain patterns: **Group 1** had a “paradoxical” strain (=positive = mechanical elongation = stretching) at 160 ms in at least a singular segment of the basal “triad” (*n* = 35) (example in Figure 1), whereas **Group 2** did not have a “paradoxical” strain (=normal) (*n* = 25) at the same 160 ms point in any of the segments of that basal “triad” (example in Figure 2; Figure 3 shows an example of a normal strain in one of the controls). The complete four-panel results are displayed as a bullseye rendering in Figure 4. The clinical characteristics were the same between **Group 1** and **Group 2** (Table 2).

Concerning **Group 1**, 19 patients showed the paradoxical strain at 160 ms in only a single segment of the basal “triad”, 10 patients in two segments, and 6 patients in all segments of the “triad”. The most involved segment was the BI observed in 27 subjects, followed by the BIL in 21 subjects, and then the BAL in 9 subjects.

**Figure 5:**
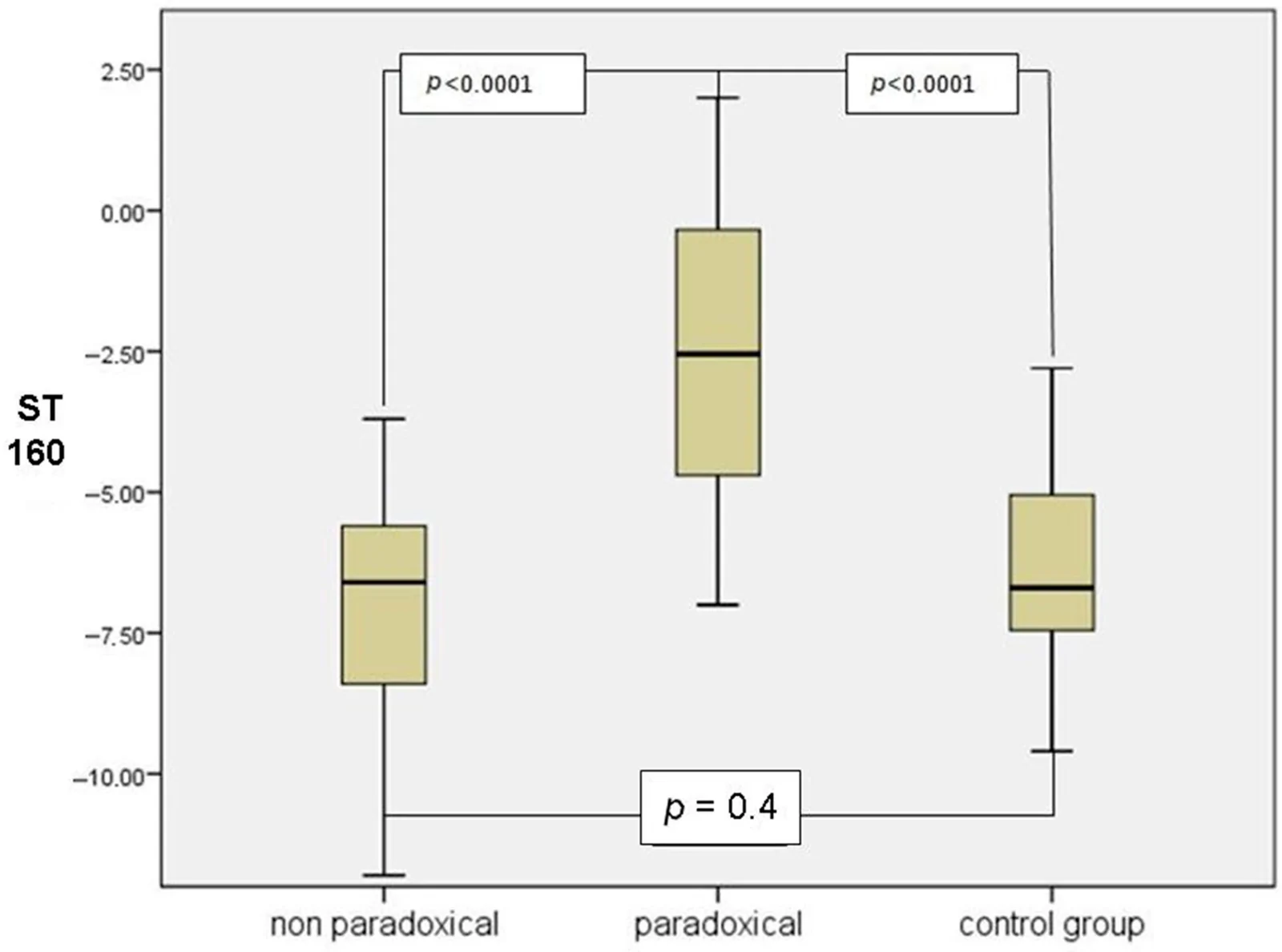
STE at 160 ms in the groups.

**Figure 6:**
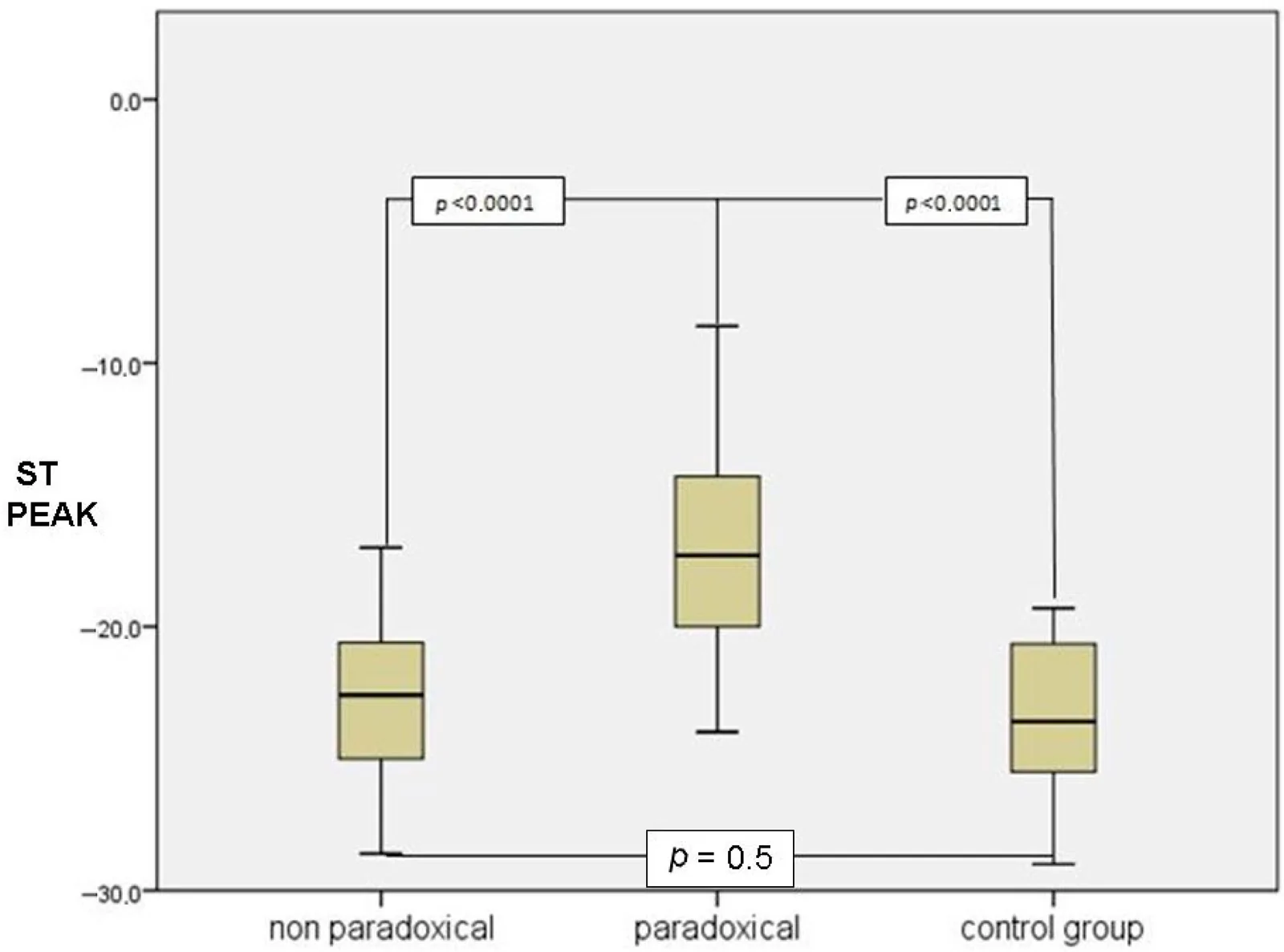
Strain in the groups at the peak.

A statistically significant difference in strain was found between the two MVP groups (**Group 1 and Group 2**) at 160 ms (Figure 5) and at the peak (*p* < 0.0001) (Figure 6), and between Group 1 and the control group at 160 ms and at the peak (*p* < 0.0001) (Figure 5 and Figure 6); on the contrary, MVP patients without paradoxical strain (**Group 2**) did not show any significant differences compared to the control group (Figure 5) (Table 3).

Concerning the GLS, we found significant differences between Groups 1 and 2 but not between Group 2 and the controls (Table 3).

Because the average STE values in the “triad” were obtained precisely by averaging the values of the strain at 160 ms in all segments of the “triad”, measured for each patient, and also because in Group 1 some patients had a paradoxical (=positive) strain in only a single segment of the “triad”, a negative (=not paradoxical, but only statistically) average value of the strain at 160 ms (−2.5) was found in Group 1 (Table 3, **line 1**).

Finally, no gross differences between the groups were found in the strain of the anterior wall; this was expected because the anterior annulus is not connected to muscles but only to the trigone and aorta (visual summary in a bullseye rendering, Figure 4).

## 4. Discussion

Mitral valve prolapse is a complex anatomical and physiological disorder in which the exact underlying mechanisms of the disease and the progression of complications remains unclear in many respects. Its clinical spectrum may vary from asymptomatic situations to a constellation of symptoms or other physical abnormalities, so that it is referred as “MVP syndrome” [5]. Although the mitral leaflets are usually the most involved structures, MVP complications can be the result of the involvement of all the other components of the mitral apparatus including the cords, the annulus, the papillary muscles and the basal inferior-lateral ventricular wall.

Patients having MVP may sometimes present themselves with cardiac arrhythmias and even (rarely) with SCD, and even without any evidence of hemodynamic impairment. Fibrosis of the papillary muscles and of the basal inferior wall have been previously described as the possible structural hallmarks of arrhythmias. However, the exact anatomical substrate of electrical instability and its role in risk stratification remain unclear.

Interestingly, Sanfilippo et al. [4] formerly observed an odd upward systolic movement of the inferior papillary muscle on echocardiography: in particular, they described an upward movement of the tip of the inferior papillary muscle toward the annulus moving away from the apex in systole (that is the opposite of normal). However, they could not further explain the cause of this abnormal papillary muscle motion due to the limited technological resources available at that time; nevertheless, they postulated that these findings might have a “concern to the mechanical and electrophysiological changes in the left ventricle” (LV).

In the present study, we visually observed an altered movement not only in the basal inferior wall but also in the basal posterior and lateral walls and then we wanted to analyze these odd wall movements with the help of STE.

Some studies showed some kinds of distinct patterns of mitral annulus excursion in healthy adults during the cardiac cycle using STE quantification: particularly, they observed that the mitral annulus excursion remained stable for a short time during the isovolumic contraction while it was rapidly moving towards the apex during the ejective systolic phase [6,7].

In the present study, we analyzed the movement of the basal wall in “the triad” (as previously described) with STE.

In our study of the MVP population, 58% of MVP patients showed a “paradoxical” strain (=positive = stretching of myocardial fibres) in at least a single segment of the basal “triad” in mid-systole, while the rest of the population (42%) had not. And so we were able to find two distinct groups of MVP patients: (1) one having a paradoxical strain in mid-systole, and (2) a second one not having a paradoxical strain in mid-systole in what we named the “triad” of basal segments.

But, we asked ourselves, what was the clinical importance of separating these two populations of MVP patients having or not having paradoxical strain in mid-systole?

We thought, the paradoxical strain of the basal segments of the inferior, anterolateral and inferolateral walls in mid-systole evidently causes continuous mechanical myocardial cell stretching. A study on MVP patients with malignant arrhythmias, undergoing Cardiac Magnetic Resonance (CMR), described an unusual systolic movement of the posterior mitral annulus on the adjacent myocardium, initially downward, which they called “systolic curling”, associated with a systolic stretch in the LV inferior-basal myocardial segments closely linked to the valve [8].

The movement of the basal segments, in a direction opposite to the normal, leads to a mid-systolic dyssynchrony. As a consequence, this might result in an early-systolic reduction in cardiac contractility, and maybe consequentially in a reduction in GLS. These mechanisms may explain our results observed in the MVP Group 1 (with paradoxical strain) compared with Group 2 (non-paradoxical strain) as we found in the between a reduction in the GLS (Table 3).

These mechanisms have also been described in a previous study carried out using a similar STE analysis, which showed an extensive abnormal strain in all LV segments of the inferior wall, especially at the basal level [9].

One important thing, in our opinion, is that the continuous stretching in mid-systole of these basal segment cells, observed in our group with paradoxical strain (**Group 1**), may also represent “per se” a cause of arrhythmias. Indeed, the continuous stretching of myocardial cells (as occurs in all excitable cells) may trigger premature electrical potentials and then it may lead to premature excitation of the cells and, in the case of the heart cells, to arrhythmias [10].

Furthermore, the continuous passive mechanical stretching of the wall (meaning the cells) in mid-systole, leads, as time goes by, to the replacement of the cells by fibrosis (and, indeed, this is the area in which fibrosis has been reported to be present by CMR studies) [8,11]. And so, these parts of the LV wall, in the presence of paradoxical strain, are doubly prone to arrhythmias: (1) because of the stretching of the cells and (2) because of the replacement by fibrosis.

The presence of this paradoxical strain in these segments in mid-systole, in our opinion, also has something to do with the presence of the so-called “Pickelhaube Sign”, which is a particular Tissue Doppler aspect of the movement of the mitral annulus that is found in some MVP patients. It appears as a positive spike in the late systole, and this is evidently partly due to the lack of muscle contraction in mid-systole (as we reported) and not only due to a simple traction by the mitral leaflet; moreover, this sign is known to be associated with arrhythmic risk, especially in the presence of a MAD (however, we excluded MVP with MAD in our study).

Finally, we wanted to make some “economical” considerations.

CMR is well known to be a very useful tool to quantify regional replacement of the wall with fibrosis in several cardiac diseases and in MVP too, and so it is able to further identify patients who are at major risk for arrhythmias, as reported [12]. However, CMR is an expensive exam which requires large medical facilities to be performed, and it cannot be performed at bedside.

On the contrary, STE analysis is a cheap tool which, by now, has also been included in the necessary “basic” evaluation of an echo exam and has to be carried out for a complete evaluation of patients in several pathologies [13].

In our opinion, according to the results of the present study, STE should be performed in MVP patients with arrhythmias in order to look for the presence of “paradoxical” STE and, if it is present, a further evaluation with CMR (even if expensive) is recommended.

Evaluating patient follow-up for arrhythmic events and the relationship with the strain were not the purpose of our study, and so we did not limit enrollment to patients who had undergone 24 h Holter monitoring or CMR. Future research performed according to a multicenter study protocol that includes Holter and CMR would be beneficial.

The outcome of MVP patients, up to now, has been mainly described as influenced by the presence of identifiable primary and secondary risk factors [1]; however, according to our results, it would be helpful to add STE analysis to the risk factors and thereby improve risk stratification.

In any case, in our opinion, in MVP patients with arrhythmias, STE is already indicated because the topic has to do with prognosis, the further study of arrhythmias, and the choice of appropriate therapy.

## Figures and Tables

**Table 1:** Clinical characteristics of MVP and control groups.

	MVP (n = 60)	Control (n = 20)	*p* Value
**Males (%)**	27 (45)	9 (45)	ns
**Females (%)**	33 (55)	11 (55)	ns
**Age (years)**	45 ± 4.3	43 ± 3.6	ns
**LVEF (%)**	68 ± 7.3	65 ± 3.5	ns
**LA LAX (mm)**	40 ± 3.9	39 ± 2.8	ns
**Prolapsing Leaflet (%)**			
**Posterior**	60 (100)	0	
**Anterior (bileaflet)**	6 (10)	0	
**BMI (kg/m^2^)**	22.1 ± 1.54	22.1 ± 1.51	ns
**Heart Rate (beats/min)**	69 ± 5.8	71 ± 6	ns
**SBP (mmHg)**	115 ± 19	112 ± 15	ns
**DBP (mmHg)**	77 ± 4	79 ± 4	ns

Table 1: Clinical characteristics of the study population: MVP and control group. No statistical differences (ns). LVEF: left ventricular ejection fraction; LA: left atrium dimension obtained in parasternal long-axis; BMI: body mass index; SBP: systolic blood pressure; DBP: diastolic blood pressure. To be noted: all patients had a posterior leaflet prolapse, even if a few (6) had both anterior and posterior prolapse.

**Table 2:** Clinical characteristics of MVP Group 1 and Group 2.

	Group 1 (n = 35)	Group 2 (n = 25)	*p*
**Male (%)**	16 (45)	11 (44)	ns
**Female (%)**	19 (55)	14 (56)	ns
**Age (years)**	47 ± 2.8	42 ± 3.2	ns
**LVEF (%)**	68 ± 6.7	65 ± 9.5	n
**LA LAX (mm)**	41 ± 3.7	39 ± 2.7	ns
**Prolapse (%)**			
**Posterior**	35 (100)	25 (100)	
**Anterior (bileaflet)**	6 (17)	0	
**MR grading (0–3)**	1	1	ns
**BMI (kg/m^2^)**	21.9 ± 1.4	22.4 ± 1.6	ns
**Heart Rate (beats/minute)**	68.3 ± 5.9	70.3 ± 5.5	ns
**SBP (mmHg)**	114.8 ± 18.4	116 ± 21	ns
**DBP (mmHg)**	78 ± 4	75 ± 5.5	ns

Table 2: Clinical characteristics between Group 1 and Group 2 showing no statistically significant differences (ns). LVEF: left ventricular ejection fraction; LA: left atrium dimensionin parasternal long-axis; BMI: body mass index; SBP: systolic blood pressure; DBP: diastolic blood pressure.

**Table 3:** Averaged strain values of the basal segments (the “triad”) for each group.

	**control group (20)**	**group 1 (35)**	***p***
ST at 160 ms	−6.3 ± 1.9	−2.5 ± 2.3	<0.0001
ST at peak	−23.4 ± 2.9	−17.0 ± 3.6	<0.0001
GLS	−20 ± 2	−18.7 ± 2.4	0.01
	**control group (20)**	**group 2 (25)**	***p***
ST at 160 ms	−6.3 ± 1.9	−7.0 ± 2	0.4
ST at peak	−23.4 ± 2.9	−22.8 ± 3.1	0.5
GLS	−20 ± 2	−20 ± 2.5	0.9
	**group 2 (25)**	**group 1 (35)**	***p***
ST at 160 ms	−7.0 ± 2	−2.5 ± 2.3	<0.0001
ST at peak	−22.8 ± 3.1	−17.0 ± 3.6	<0.0001
GLS	−20 ± 2.5	−18.7 ± 2.4	0.01

Table 3: Mean strain values at 160 ms and at peak for the basal segments of the “triad” in each group. Statistical significance was found at 160 ms between Group 1 and the controls, between Group 2 and Group 1, and for GLS. ST: strain. GLS: Global Longitudinal Strain.

## Data Availability

The data presented in this study are openly available at https://doi.org/10.1093/ehjci/jeae333.094.
